# Supplementary material for: Cohesin distribution alone predicts chromatin organization in yeast via conserved-current loop extrusion
Source: Genome Biol. 2024 Nov 14;25:293. doi: 10.1186/s13059-024-03432-2 (PMC11566905; doi:10.1186/s13059-024-03432-2)
Supplement: Supplementary file 2 — Additional file 2. Table of cited experimental datasets. [file 13059_2024_3432_MOESM2_ESM.docx]

Table S2. Information of datasets used in the manuscript.

| Organism/cell stage/cell type  (wildtype if not specified) | Data content | First Author (and DOI in parentheses) | GEO accession number | Dataset file name/SRR accession | Acquired data format | Usage in figures |
| --- | --- | --- | --- | --- | --- | --- |
| *S. pombe* (Interphase) | Psc3 ChIP-seq | Mizuguchi *et al.* (10.1038/nature13833) | GSE56849: GSM1370192 | GSM1370192_SG12054172_  251601010199_S001_ChIP_  1100_Jul11_1_2 | .txt | 1, 3, S1-S9, S11, S19, S20, S21 |
| *S. pombe* (Interphase) | Hi-C |  | GSE56849: GSM1379427 | GSM1379427_wt_999a-corrected-matrix_hic | .tsv | 1, 2, S1-S9, S11, S15 |
| *S. cerevisiae*  (Meiotic) | Rec8 ChIP-seq | Ito *et al.* (10.1111/gtc.12138) | GSE52863: GSM1277163 | GSM1277163_Rec8.wt.  4h.saccer2 | .bedgraph | 5, S18 |
| *S. cerevisiae*  Meiotic | Hi-C | Schalbetter *et al.* (10.1038/s41467-019-12629-0) | GSE127940: GSM3656898 | GSM3656898_HiC_SSY14_ndt80D_1A1_8h_2000_iced | .matrix | 5 |
|  |  |  | GSE127940: GSM3656899 | GSM3656899_HiC_SSY14_ndt80D_2A2_8h_2000_iced |  |  |
| *S. cerevisiae* (Mitotic) | Mcd1 ChIP-seq | Costantino *et al.* (10.7554/eLife.59889) | GSE151416: GSM4577764 | GSM4577764_Mcd1p_WT | .bigwig | 6, S14, S18, S22, S23 |
|  |  |  | GSE151416: GSM4577765 | GSM4577765_Mcd1p_WT_2 |  |  |
| *S. cerevisiae*  (Mitotic) | Hi-C |  | GSE151553: GSM4585126 | SRR11893084 | .FASTQ | 6 |
|  |  |  | GSE151553: GSM4585127 | SRR11893085 |  |  |
| *S. pombe*  (Interphase) | Micro-C XL | Hsieh *et al.* (10.1038/nMeth.4025) | GSE85220: GSM2262340 | SRR4000682 | .FASTQ | S5-S9, S20 |
| *S. pombe*  (Interphase) | Cut14 ChIP-seq | Nakazawa *et al.* (10.1111/gtc.12239) | GSE65956 | GSE65956_AS_Cut14-3FLAG.  w500.b10.pseuduvalue10.Ratio. | .wig | S11 |
| *S. cerevisiae* (meiotic) | Hi-C | Barton *et al.* (10.7554/eLife.74447) | GSE185021: GSM5603468 | SRR16118284 | .FASTQ | S17 |
| *S. cerevisiae* (meiotic) | Smc3 ChIP-seq |  | GSE185021: GSM5603484 | GSM5603484_IP-Smc3_28719_wt_SK1_calibrated | .bigwig | S17 |
|  |  |  | GSE185021: GSM5603492 | GSM5603492_IP-Smc3_29315_wt_SK1_calibrated |  |  |
| *S. cerevisiae* (meiotic)  Wpl1-depleted | Hi-C |  | GSE185021: GSM5603470 | SRR16118282 | .FASTQ | S17 |
| *S. cerevisiae* (meiotic)  Wpl1-depleted | Smc3 ChIP-seq |  | GSE185021: GSM5603486 | GSM5603486_IP-Smc3_29750_  wpl1_SK1_calibrated | .bigwig | S17 |
|  |  |  | GSE185021: GSM5603494 | GSM5603494_IP-Smc3_30310_  wpl1_SK1_calibrated |  |  |
| *S. cerevisiae* | Gene sequences | *Saccharomyces*Genome Database (10.1093/genetics/iyab224) | N/A | N/A | .gff3 | S18 |
| *S. pombe* | Gene sequences | Pombase (10.1093/genetics/iyae007) | N/A | N/A | .gff3 | S19 |
| *S. pombe*  (Interphase) | Rad21 ChIP-seq | Nakazawa *et al.* (10.1111/gtc.12239) | GSE65956 | GSE65956_AS_Rad21-3FLAG.  w500.b10.pseuduvalue10.Ratio | .wig | S21 |
| *S. pombe*  (Interphase) | Mis4 ChIP-seq | Schmidt *et al.* (10.1186/gb-2009-10-5-r52) | GSE13517: GSM333163 | Data table | Data table | S21 |
| *S. pombe*  (Interphase) | Pds5 ChIP-seq |  | GSE13517: GSM333006 | Data table | Data table | S21 |
| *H. sapiens* | Smc1 ChIP-seq | Garcia *et al.* (10.1038/s41467-021-24808-z) | GSE145966: GSM4340358 | GSM4340358_SMC1A_Control | .bedgraph | S21 |
| *H. sapiens* | Nipbl ChIP-seq |  | GSE145966: GSM4340364 | GSM4340364_NIPBL_Control | .bedgraph | S21 |
| *S. cerevisiae*  (Mitotic) | Pds5 ChIP-seq | Chapard *et al.* (10.1016/j.molcel.2019.05.023) | GSE120138: GSM3394810 | GSM3394810_5IP | .bigwig | S22 |
| *S. cerevisiae*  (Mitotic) | Scc2 ChIP-seq | Mattingly *et al.* (10.1016/j.cub.2022.05.019) | GSE201193: GSM6052391 | GSM6052391_scc2_noIAA_1_ peaks | .narrowpeak | S22, S23 |
