# Supplementary material for: Cohesin distribution alone predicts chromatin organization in yeast via conserved-current loop extrusion
Source: Genome Biol. 2024 Nov 14;25:293. doi: 10.1186/s13059-024-03432-2 (PMC11566905; doi:10.1186/s13059-024-03432-2)
Supplement: Supplementary file 3 — Additional file 3. Review history. [file 13059_2024_3432_MOESM3_ESM.docx]

Review History

**First round of review**

**Reviewer 1**

**Comments to author:**

**Review Commons *Report 1*.** [**https://doi.org/10.15252/rc.2024971669**](https://doi.org/10.15252/rc.2024971669)

**Reviewer 2**

**Comments to author:**

**Review Commons *Report 2*.** [**https://doi.org/10.15252/rc.2024860940**](https://doi.org/10.15252/rc.2024860940)

**Authors Response**

**Point-by-point responses to the reviewers’ comments:** [**https://doi.org/10.15252/rc.2024133506**](https://doi.org/10.15252/rc.2024133506)

**Second round of review**

**Reviewer 1**

=The manuscript is somewhat improved but the major issue with this work is that the authors draw strong conclusions from the modelling that is not always supported by published experimental data. The value of a model that recapitulates genome structure based on cohesin patterns is appreciated, but to be useful the caveats must be pointed out. The wider literature should be considered before drawing conclusions based soley on the model. Since this is just a model, the authors should be more nuanced in their conclusions and where the model does not fit the experiment, they should not simply dismiss the experimental data as "noisy". In addition, there are other and more recent and very high quality experimental datasets published which have not been analysed. If, as the authors argue, the lack of agreement between the model and the experiments is noisy data, then this issue should be solved by looking at less noisy data. Until this has been done, conclusions based on the model, rather than experiments should not be made so strongly. Key points that should be addressed are:

(1) The argument about the requirement of Scc2/Nipbl for loop extrusion raised by reviewer 2 is a very good one and in addressing this point the authors have ignored/dismissed key biochemical data. The understanding in the field is that Scc2/Nipbl drives loop extrusion both in vivo and in vitro in yeast and mammals. It is not simply a loading factor as indicated by the authors. Why it is not detected at cohesin sites by ChIP-Seq is not clear but it could be a technical issue related to ChIP-Seq and residency times. What is clear is that Scc2 stimulates cohesin's ATPase activity and loop extrusion in vitro and that degradation of Scc2 prevents loop extrusion in vivo (Bastié NSMB 2022).

(2) In the original review, it was suggested to example datasets lacking Wpl1. This data is available in Barton (eLife 2022) (GSE185021). There are also Hi-C datasets for meiotic prophase with high resolution.

(3) The limitations of the model need to be more clearly stated throughout. The tone of the manuscript is strongly endorsing the model throughout, while there are clear limitations that should be stated to avoid misleading the reader.
(4) As pointed out in the previous reviews, the diffusion capture model is largely discounted in the field where the general consensus is that loop extrusion defines genome organisation. The idea that the current work resolves some major controversy in this regard should be toned down as this is not the case.

(5) Line 165 "by eye, we judge the agreement displayed in Figs. 1A and B to be comparable or superior". Such subjective comparisons should be avoided. If the authors want to make this comparison they should perform quantitative analysis as in Figure 1E and F. Could the authors also make a single numeric value from these plots as a measure of fit for each comparison?

(6) Supplementary Figures 5-9. The authors argue that the poor correlation between the model prediction and the micro-C data from S. pombe is due to the data being noisy. However, there is a lot of detail in the experimental data at high resolution that is missed by the model and a different and more straightforward interpretation of this data is that the model is not good at predicting the data. Therefore, the statements around this need to be toned down and it should be explicitly stated in the abstract and all conclusions that the model does a good job in the 10-100kb range but fails to predict organisation at smaller scales/resolution. The same is true for the budding yeast mitotic data.

(7) Line 415 "The lack of cohesin background signal in the mitotic case likely suggests that the mitotic cohesive cohesin density is very low, if not completely zero". This cannot be the case because chromosomes are held together in a cohesin-dependent manner. It is well established that cohesin density cannot predict where cohesive cohesin resides. It has also previously been shown that cellular cohesin levels can be greatly reduced without impacting cohesion, indicating that the majoriy of cohesin on chromosomes is not cohesive (Heidinger-Pauli Curr Biol 2010).

**Reviewer 2**

The revised manuscript by Yuan et al. has largely addressed the critiques raised after the initial submission. The model is broadly interesting and informative, and technically, the work is sound. The authors have adequately revised the presentation of the model and the ensuing discussion to better fit the results and analysis. The revision also includes substantial new work including analysis of convergent gene positioning, simulations of an alternative model (diffusion-capture), and additional analysis and modeling of yeast data. Although I disagree with a few statements in the manuscript (and more in the rebuttal), the manuscript has generally been revised to fall within the realm of reasonable disagreement and discussion. This new approach to modeling loop extrusion adds to the considerable evidence in favor of loop extrusion in the literature, particularly in yeast where there has been comparably less modeling of how cohesin might generate TAD-like domains, and the methodology is in principle applicable to other systems. I believe this manuscript is suitable for Genome Biology, but I have a two remaining comments that should be addressed.

1. Regarding the assumption of binding and unbinding rates being small (or balanced) compared to left/right currents: The authors discuss current violations in models of yeast data, and argued that these violations are small and/or localized. However, a stated motivation for the work is to construct a more general fundamental and broadly applicable physical model for extrusion. So, while I do not really question the goodness of the model for the yeast data analyzed here, I wonder about the extent to which CCLE with the stated assumptions can be applied to other genomes which might have strong and/or widespread loading or unloading sites. Generally, the authors argue that the assumptions are justified a posteriori because the model agrees with the data; this is okay for the yeast data, but does CCLE always work by a posteriori justification? For example, in an unfamiliar organism, if we just have cohesin ChIP-seq but no Hi-C, can we trust the predicted Hi-C from CCLE? At the end of the results, the authors suggest that there is a way to handle current violations. A few more words on this issue would be helpful to more clearly explain the generality and/or limitations of CCLE.

2. Regarding the new comparison to the explicit barrier model of Schalbetter et al.: while it is beneficial for the authors to compare the CCLE results to those of alternative models, I do not think it is necessarily a fair comparison for the authors to implement best fit values (for extrusion passing through barriers) derived from the explicit barrier model in S. cerevisiae into a model designed to replicate data from S. pombe. It may be that this is the best that can be done without the large (and unreasonable) burden of entirely recapitulating the simulations of Schalbetter et al. (I do not know). The authors should provide a rationale for the use of this parameter value, acknowledge this limitation of the comparison, or find the actual best fit for S. pombe and make a comparison between CCLE and explicit barriers with that best fit.

**Authors Response**

**Point-by-point responses to the reviewers’ comments:**

*Reviewer #1: =The manuscript is somewhat improved but the major issue with this work is that the authors draw strong conclusions from the modelling that is not always supported by published experimental data. The value of a model that recapitulates genome structure based on cohesin patterns is appreciated, but to be useful the caveats must be pointed out. The wider literature should be considered before drawing conclusions based soley on the model. Since this is just a model, the authors should be more nuanced in their conclusions and where the model does not fit the experiment, they should not simply dismiss the experimental data as "noisy". In addition, there are other and more recent and very high quality experimental datasets published which have not been analysed. If, as the authors argue, the lack of agreement between the model and the experiments is noisy data, then this issue should be solved by looking at less noisy data. Until this has been done, conclusions based
on the model, rather than experiments should not be made so strongly. Key points that should be addressed are:*

*(1) The argument about the requirement of Scc2/Nipbl for loop extrusion raised by reviewer 2 is a very good one and in addressing this point the authors have ignored/dismissed key biochemical data. The understanding in the field is that Scc2/Nipbl drives loop extrusion both in vivo and in vitro in yeast and mammals. It is not simply a loading factor as indicated by the authors. Why it is not detected at cohesin sites by ChIP-Seq is not clear but it could be a technical issue related to ChIP-Seq and residency times. What is clear is that Scc2 stimulates  cohesin's ATPase activity and loop extrusion in vitro and that degradation of Scc2 prevents loop extrusion in vivo (Bastié NSMB 2022).*

We thank the referee for pointing out Bastié et al., which we now cite in the revised manuscript. In response to this comment, we have calculated the cross correlation between the ChIP-seq data of a subunit of cohesin core complex, Mcd1, and Scc2, in non-centromeric regions of all chromosomes in mitotic *S. cerevisiae*. None of the regions shows significant cross correlations above the background level. This observation for mitotic *S. cerevisiae* suggests that the activity of Scc2 in driving DNA loop expansion does not occur through simple colocalization or co-translocation with the cohesin core. However, in our view, the absence of cohesin-Scc2 correlation and the absence of cohesin-Scc2 colocalization do not preclude the possibility of an Scc2-mediated translocation process driving expansion of DNA loops, as argued by Bastié *et al*. Scc2 could interact indirectly via recruitment of currently-unknown factors, for example. We have modified the corresponding paragraph in the Discussion section of the revised manuscript (lines 589-612) to reflect the above discussion/comment.

*(2) In the original review, it was suggested to example datasets lacking Wpl1. This data is available in Barton (eLife 2022) (GSE185021). There are also Hi-C datasets for meiotic prophase with high resolution.*

We thank the referee for providing the information about Hi-C and ChIP-seq data of Wpl1-depleted budding yeast strain. We have run CCLE simulations for the Wpl1-depleted data and its wild-type counterpart and have obtained the corresponding simulated Hi-C maps (new Supplementary Fig. 17). In both cases, the CCLE-simulated Hi-C maps agree well with the experimental Hi-C data. Furthermore, the comparison between the best-fit simulated Hi-C maps using wildtype and Wpl1-depleted cohesin ChIP-seq data suggests that our CCLE model can indeed predict an increased range of chromatin-chromatin contacts and an increased strength of loop anchors in the absence of Wpl1 (new Supplementary Fig. 17). This new supplementary figure and the corresponding added discussion (lines 413-417) fully respond to this comment by the referee.

*(3) The limitations of the model need to be more clearly stated throughout. The tone of the manuscript is strongly endorsing the model throughout, while there are clear limitations that should be stated to avoid misleading the reader.*

The assumptions made by the model are clearly stated in lines 123-126 of the submitted manuscript, and the approaches to quantify the violations of these assumptions are provided in lines 266-275 of the submitted manuscript. The quantifications of these violations for all three cases, namely interphase *S. pombe*, meiotic *S. cerevisiae*, and mitotic *S. cerevisiae*, are presented in Supplementary Figures 12, 13, and 14 of the submitted manuscript, and are mentioned in each corresponding section.

Given that the point of our manuscript is to introduce, describe, and disseminate the CCLE model, of course, the manuscript endorses the CCLE model. The referee is correct that the CCLE model does not describe chromatin organization at the nucleosomal scale, which has recently been shown to be manifest in high-resolution Hi-C maps at kilobase genomic separations (Ohno et al. Cell 176(3):520-34 2019, Wiese et al. PNAS 116(35):17307-15 2019, Oberbeckmann et al. Nat. Genet. 56(3):483-92 2024). In the revised manuscript, we have now added a discussion of these results (lines 53-55) and have clearly stated that CCLE does not account for nucleosomal-scale contacts (lines 108-110).

*(4) As pointed out in the previous reviews, the diffusion capture model is largely discounted in the field where the general consensus is that loop extrusion defines genome organisation. The idea that the current work resolves some major controversy in this regard should be toned down as this is not the case.*

Multiple recent studies including Gerguri et al. Nucleic Acids Res. 49(3):1294-312 2021, Tang et al. Mol. Cell 83(21):3787-800 2023, and Conte et al. Nat. Commun. 13(1):4070 2022, continue to favor a diffusion capture model over loop extrusion. Therefore, we disagree with the referee that the diffusion capture model is “largely discounted in the field”. Nowhere in the manuscript do we claim that we have resolved a major controversy between loop extrusion and diffusion capture, nor does the referee offer any specific suggestions or requests concerning how to “tone down” the manuscript in this regard. Moreover, the discussion in the manuscript concerning diffusion capture was added specifically in response to Review Commons Referee #2’s request that we investigate a version of the diffusion capture model that is consistent with cohesin ChIP-seq, which we now do. Therefore, we prefer not to remove or change this discussion in the submitted manuscript.

*(5) Line 165  "by eye, we judge the agreement displayed in Figs. 1A and B to be comparable or superior". Such subjective comparisons should be avoided. If the authors want to make this comparison they should perform quantitative analysis as in Figure 1E and F. Could the authors also make a single numeric value from these plots as a measure of fit for each comparison?*

For every experiment-simulation Hi-C comparison in our manuscript, including Figures 1A and B, we have calculated both the MPR and the Pearson Correlation Coefficient as quantitative metrics of the quality of our simulated Hi-C maps. For Figures 1A and B, these quantities are given in Table 1. Because the quantitative metrics are provided in the manuscript, we do not think it is objectionable to continue to express our subjective opinion that CCLE provides a comparable or superior description of experimental Hi-C maps to other models.

*(6) Supplementary Figures 5-9. The authors argue that the poor correlation between the model prediction and the micro-C data from S. pombe is due to the data being noisy. However, there is a lot of detail in the experimental data at high resolution that is missed by the model and a different and more straightforward interpretation of this data is that the model is not good at predicting the data. Therefore, the statements around this need to be toned down and it should be explicitly stated in the abstract and all conclusions that the model does a good job in the 10-100kb range but fails to predict organisation at smaller scales/resolution. The same is true for the budding yeast mitotic data.*

As noted above, the referee is correct that the CCLE model does not describe chromatin organization at the nucleosomal scale, which has recently been shown to be manifest in high-resolution Hi-C maps at kilobase genomic separations (Ohno et al. Cell 176(3):520-34 2019, Wiese et al. PNAS 116(35):17307-15 2019, Oberbeckmann et al. Nat. Genet. 56(3):483-92 2024). In the revised manuscript, we have now added a discussion of these results (lines 53-55) and have clearly stated that CCLE does not account for nucleosomal-scale contacts (lines 108-110).

*(7) Line 415 "The lack of cohesin background signal in the mitotic case likely suggests that the mitotic cohesive cohesin density is very low, if not completely zero". This cannot be the case because chromosomes are held together in a cohesin-dependent manner. It is well established that cohesin density cannot predict where cohesive cohesin resides. It has also previously been shown that cellular cohesin levels can be greatly reduced without impacting cohesion, indicating that the majoriy of cohesin on chromosomes is not cohesive (Heidinger-Pauli Curr Biol 2010).*

We agree with the referee that the cohesive cohesin density cannot be zero. Therefore, we have removed the phrase “if not completely zero” in the revised manuscript. *Reviewer #2: The revised manuscript by Yuan et al. has largely addressed the critiques raised after the initial submission. The model is broadly interesting and informative, and technically, the work is sound. The authors have adequately revised the presentation of the model and the ensuing discussion to better fit the results and analysis. The revision also includes substantial new work including analysis of convergent gene positioning, simulations of an alternative model (diffusion-capture), and additional analysis and modeling of yeast data. Although I disagree with a few statements in the manuscript (and more in the rebuttal), the manuscript has generally been revised to fall within the realm of reasonable disagreement and discussion. This new approach to modeling loop extrusion adds to the considerable evidence in favor of loop extrusion in the literature, particularly in yeast where there has been comparably less modeling of how cohesin might generate TAD-like domains,
and the methodology is in principle applicable to other systems. I believe this manuscript is suitable for Genome Biology, but I have a two remaining comments that should be addressed.

1. Regarding the assumption of binding and unbinding rates being small (or balanced) compared to left/right currents: The authors discuss current violations in models of yeast data, and argued that these violations are small and/or localized. However, a stated motivation for the work is to construct a more general fundamental and broadly applicable physical model for extrusion. So, while I do not really question the goodness of the model for the yeast data analyzed here, I wonder about the extent to which CCLE with the stated assumptions can be applied to other genomes which might have strong and/or widespread loading or unloading sites. Generally, the authors argue that the assumptions are justified a posteriori because the model agrees with the data; this is okay for the yeast data, but does CCLE always work by a posteriori justification? For example, in an unfamiliar organism, if we just have cohesin ChIP-seq but no Hi-C, can we trust the predicted Hi-C from CCLE? At the
end of the results, the authors suggest that there is a way to handle current violations. A few more words on this issue would be helpful to more clearly explain the generality and/or limitations of CCLE.*

We thank the referee for raising this point as self-consistently accounting for violations of assumptions is essential for CCLE’s greater generality and robustness in its predictive power. We have outlined, at the end of the Results section (lines 479-486), the procedures by which we can improve the CCLE model in the future.

*2. Regarding the new comparison to the explicit barrier model of Schalbetter et al.: while it is beneficial for the authors to compare the CCLE results to those of alternative models, I do not think it is necessarily a fair comparison for the authors to implement best fit values (for extrusion passing through barriers) derived from the explicit barrier model in S. cerevisiae into a model designed to replicate data from S. pombe. It may be that this is the best that can be done without the large (and unreasonable) burden of entirely recapitulating the simulations of Schalbetter et al. (I do not know). The authors should provide a rationale for the use of this parameter value, acknowledge this limitation of the comparison, or find the actual best fit for S. pombe and make a comparison between CCLE and explicit barriers with that best fit.*

In response to the referee’s concern about using the best-fit parameter of the pass-through probability of meiotic *S. cerevisiae* in interphase *S. pombe*, we have performed additional simulations in the context of interphase *S. pombe*, using a wide range of pass-through probability values from 0.005 to 0.5. We observed that within this hundred-fold variation range the pass-through probability is insignificant to the simulation results, illustrated in the revised Supplementary Fig. 20. We have also provided the rationale for using this parameter value in the revised manuscript (lines 532-535).

**Third round of review**

**Reviewer 1**

The authors have addressed the comments from the previous revision in a satisfactory manner. One outstanding issue is that the datasets used for the experimental data and their original source are difficult to find and it some cases do not appear to be cited. Can the authors provide a table which details the datasets used? The authors should also ensure they have cited the original studies where the datasets where generated in the main text.

**Reviewer 2**

The authors have addressed my comments from the previous report, but I identify two issues with the revision, particularly in changes made attempting to address the other Referee. Although I appreciate the value of the model introduced by manuscript, the issue of over-interpretation and overstating conclusions persists in the discussion & analysis of the ChIP-seq.

1. Somewhat major issue. In my view, the newly added text in lines 590-593 goes beyond what could be reasonably interpreted from existing experiments and the new analysis (“The absence of correlation between the ChIP-seq of Scc2 and cohesin core in mitotic S. cerevisiae suggests that the activity of Scc2 in driving DNA loop expansion involves a mechanism other than colocalization or co-translocation with the cohesin core.”). As I pointed out in my original review, and noted again previously by Referee 1, technical issues with ChIP-seq are a major suspect here. I do not think the lack of correlation above background is sufficient to rule out or suggest ruling out colocalization/cotranslocation mechanisms. Furthermore, I do not understand what the authors mean by mechanism other than colocalization mechanism and what that would look like; e.g., do the authors mean that Scc2 does not directly interact with cohesin (in contrast to many previous experiments)? Therefore, I do not think the authors have adequately addressed this issue.

2. Minor issue. Lines 432-442. While I agree with the authors that higher res Hi-C will be noisier, at the individual pixel level, the authors should acknowledge that there are likely other experimental & physiological details not captured by the CCLE model that can lead to lower MPR and PCC for different experiments and cell types.

**Authors Response**

**Point-by-point responses to the reviewers’ comments:**

*Reviewer #1: The authors have addressed the comments from the previous revision in a satisfactory manner. One outstanding issue is that the datasets used for the experimental data and their original source are difficult to find and it some cases do not appear to be cited. Can the authors provide a table which details the datasets used? The authors should also ensure they have cited the original studies where the datasets where generated in the main text.*

Such a table is provided in Additional File 2: Table S2. All original studies where the datasets were generated are also cited in the main manuscript.

*Reviewer #2: The authors have addressed my comments from the previous report, but I identify two issues with the revision, particularly in changes made attempting to address the other Referee. Although I appreciate the value of the model introduced by manuscript, the issue of over-interpretation and overstating conclusions persists in the discussion & analysis of the ChIP-seq.

1. Somewhat major issue. In my view, the newly added text in lines 590-593 goes beyond what could be reasonably interpreted from existing experiments and the new analysis ("The  absence of correlation between the ChIP-seq of Scc2 and cohesin core in mitotic S. cerevisiae suggests that the activity of Scc2 in driving DNA loop expansion involves a mechanism other than colocalization  or co-translocation with the cohesin core."). As I pointed out in my original review, and noted again previously by Referee 1, technical issues with ChIP-seq are a major suspect here. I do not think the lack of correlation above background is sufficient to rule out or suggest ruling out colocalization/cotranslocation mechanisms. Furthermore, I do not understand what the authors mean by mechanism other than colocalization mechanism and what that would look like; e.g., do the authors mean that Scc2 does not directly interact with cohesin (in contrast to many previous experiments)? Therefore, I do not think the authors have adequately addressed this issue.*

We have softened our argument regarding correlation between ChIP-seq data. We have also elaborated on what we mean by mechanism other than (persistent) colocalization and co-translocation. The referenced text by the Referee now reads:

“Assuming that ChIP-seq data correctly reflects cohesin distribution, the absence of correlation between the ChIP-seq of Scc2 and cohesin core in mitotic *S. cerevisiae* suggests that the activity of Scc2 in driving DNA loop expansion involves a mechanism other than persistent colocalization or co-translocation with the cohesin core. However, this observation does not rule out the possibility that Scc2 directly interacts with the cohesin core. Indeed, as a commonly-identified cohesin loading complex, Scc2 could transiently colocalize with the cohesin core as loading takes place.” *2. Minor issue. Lines 432-442. While I agree with the authors that higher res Hi-C will be noisier, at the individual pixel level, the authors should acknowledge that there are likely other experimental & physiological details not captured by the CCLE model that can lead to lower MPR and PCC for different experiments and cell types.*

We have added the following sentence after the referenced text by Referee to highlight the scope of CCLE and its limitation in describing high-resolution data.

“It is important to note that the poorer MPR and PCC scores could also arise from the kilobase-scale features that originate from nucleosomes in high-resolution Hi-C maps, as CCLE does not describe nucleosome-level organization.”
